# Shame, stigma and medicine

**DOI:** 10.1136/medhum-2017-011392

**Published:** 2017-12-01

**Authors:** Barry Lyons, Luna Dolezal

**Affiliations:** 1 Bioethics, School of Medicine, University of Dublin Trinity College, Dublin, Ireland; 2 Centre for Cultures and Environments of Health, University of Exeter, Exeter, UK

**Keywords:** medical humanities

“As a young physician in the mid-80s, caring for people who had contracted HIV, I lost two of my patients to suicide at a time when the virus was doing very little harm to them. I have always thought of them as having been killed by a metaphor, by the burden of secrecy and shame associated with the disease”.

         Abraham Verghese^1^

When, in the late 1980s, the psychiatrist Donald Nathanson organised a symposium on the nature of shame, it turned out to be the first such event to deal with the subject in the history of psychiatry or psychoanalysis on either side of the Atlantic. It seems strange that such a ubiquitous emotion had excited so little academic interest to that point, but as Nathanson points out—shame makes us so uncomfortable that we will go to great lengths to avoid it.^2^ Yet, despite our efforts to ignore it, shame remains universal—there can be few of us who have not been seared by shame at some stage of our life. In fact, some philosophers argue that shame is inescapable in human experience, a fundamental part of child development that textures personal, social and political aspects of adolescent and adult life.^3 4^ As Tanner states (when responding to an accusation of impudence) in Shaw’s *Man and Superman*:

“Yet even I cannot wholly conquer shame. We live in an atmosphere of shame. We are ashamed of everything that is real about us; ashamed of ourselves, of our relatives, of our incomes, of our accents, of our opinions, of our experience, just as we are ashamed of our naked skins”.^5^

The prevalence of shame in the medical clinic was identified by the physician Aaron Lazare around the same time as Nathanson’s symposium. Lazare proposed that patients were at a high risk of humiliation and shame as a consequence of their illness, and their physical and psychological exposure in the doctor’s office. However, doctors themselves are not immune to shame, and Lazare identified a number of circumstances (most notably medical failure) where clinicians may also be particularly vulnerable.^6^ However, with the exception of the field of psychology, little conceptual or empirical work has built on these foundations. One explanation for this deficit may lie in the marginal status emotional reactions have within Western medicine, and a consequent tendency to relegate and classify such responses as an irrational subtext. Danielle Ofri’s analysis of the emotional dimension of medical practice argues that contemporary perspectives often reinforce the traditional image of the rational, unwavering doctor resolutely focused on investigation, diagnosis, prognosis, decision-making and treatment.^7^ This leaves little scope for exploration of the fear, anxiety, guilt, anger, compassion, empathy or shame that many patients and practitioners may routinely experience. It remains the case that a clinician exposing such an emotional inner self may be thought of as weak, or perhaps even unprofessional, notwithstanding the matter that what doctors feel might well impact on the kind or quality of medical care delivered, not least through the possible counter-humiliation of the patient by the shamed doctor.

There is an expectation that patients should also be robust. ‘You must be very tough. Do you think you can be very tough?’ asks Dr Kelekian of his patient Vivian Bearing in Margaret Edson’s drama, *W;t*.^8^ Many illnesses (although most notably cancer) are characterised by people ‘fighting’ or being ‘at war’ with their condition. Such a narrative makes it difficult to admit to feeling shamed by disease, disfigurement or disability. A further impediment to the investigation of shame that is shared by doctors and patients is that neither are likely to wish to discuss the matter—as Lazare put it: ‘it is shameful and humiliating to admit that one has been shamed and humiliated’.^6^ As the passage from Verghese which opens this editorial demonstrates, the burden of secrecy about shame can be highly pernicious, even deadly.^1^

The *Shame and Medicine Project* 9 evolved from a conversation between the two editors of this Special Issue of *Medical Humanities* about the politics of shame and humiliation within healthcare. One is a philosopher with an academic focus in the phenomenology of the body, the other a clinician with an interest in medical training and regulation. Over the 2 years the project has run thus far, we have had the pleasure of hearing from scholars and practitioners from over 20 distinct and diverse disciplines—from theology and history to plastic surgery and contemporary visual culture. It has been a truly interdisciplinary engagement and one that has reflected shame’s role as a commonplace social emotion, frequently ‘underground’ or hidden,^10^ but which has affected, and continues to impact on, many health-related interactions between individuals and society, patients and doctors, clinicians and regulators. Shame is the primary ‘emotion of politics and conformity’ particularly, as Myra Mendible points out in *American Shame*, ‘for those who have the ‘wrong’ bodies or the ‘wrong’ desires’.^11^ Those who deviate from entrenched social norms are frequently subjected to stigma—‘a social process, experienced or anticipated, characterised by exclusion, rejection, blame or devaluation that results from experience, perception or reasonable anticipation of an adverse social judgement about a person or group’.^12^ Stigma, as many of the articles in this Special Issue demonstrate, is intimately bound up with shame. In moments of stigma, Goffman famously noted, ‘shame becomes a central possibility’.^13^ Stigma is a social, emotional, political and clinical issue of enormous significance—the impact of social exclusion contributes substantially to the burden of illness, perhaps to the extent that in highly stigmatised disorders the suffering brought on by the disease process may be outweighed by the impact of stigma-induced social rejection.

This Special Issue seeks to elaborate on the themes of shame, stigma and medicine from a variety of disciplinary perspectives and experiential angles—exploring the shame and stigma experienced by patients in illness and the shame that frequently befalls the healthcare professional. The articles collected here tackle these issues from both historical and contemporary perspectives. One area analysed from both viewpoints in this issue is that of infectious disease. Hutchinson and Dhairyawan offer a philosophical reflection on HIV and the negative impact shame may have for a person living with HIV particularly in respect of their engagement with, or retention in, the therapeutic process. HIV shares characteristics with other forms of chronic illness in that those who have been infected with the virus have a necessary (perhaps dishonourable) dependence on healthcare and maintenance medication. But HIV carries a greater moral load than just chronicity and reliance. HIV ‘serves as a vector through which pass many of a society’s existing prejudices’. HIV-stigma arises from bias and bigotry premised on negative attitudes to sex and sexuality in the context of suppositions and judgments about the ‘normal’ and the ‘perverse’, the ‘clean’ and the ‘dirty’.^14^

Jane Megan Northrop carries on the subject of infectivity stigma through a sociological evaluation of qualitative data gathered from people who had tested positive for the hepatitis C virus (HCV). Again, the association of HCV and socially ‘problematic’ behaviours led to a need for concealment in order to minimise impact on personal relationships, employment opportunities and access to health services. Participant narratives outlined senses of shame, fears of potential stigmatisation, and actual stigmatisation experienced in the healthcare setting. The stories echo the work of Hutchinson and Dhairyawan—those carrying the virus being seen as ‘agents of contagion’ with the word ‘dirty’ a constant refrain in the discourse: a ‘dirty disease’ or a ‘dirty little secret’, being placed on ‘dirty’ lists for surgery; their blood ‘polluted and perilous’, sheets burned following discharge from hospital.^15^

Of course, shame and contagion are hardly new bedfellows. These papers are balanced by Emily Cock’s article which offers a historical evaluation of shame in medical practice through the prominent 18th century surgeon, Daniel Turner. In his treatise, *Syphilis. A Practical Dissertation on the Venereal Disease* (1717), Turner concurred with the belief that the pox represented a personal punishment for sexual sins, ‘condemning a female patient’s genitals as “fittest to suffer on Account of the wanton Use she had made of them” ’.^16^ However, what is particularly interesting about Turner from a contemporary perspective is his use of shaming as a therapeutic tool, a means of compelling patients to seek and comply with treatment, to get better in order to return to a functional social role.

Medical compliance may mean more than simply following the doctor’s orders—sometimes it might entail engagement with the ‘medical model’ in the first place. Janice McLaughlin’s investigation of medical treatment as ‘a route to normality’ explores the use of medical procedures by young disabled people to reshape their bodies in order to allow for more ‘normal’ social interaction and embodiment.^17^ Medicine problematically configures social perspectives of the normal body through its willingness or desire to ‘fix’, with the contemporary sociopolitical focus on self-sufficiency and self-care contributing an important backdrop. In this context, repeated (usually painful) interactions with medicine become a management strategy through which disabled young people negotiate the social penalties attached to disability-stigma, a labour undertaken to minimise the interactional discomfort of others.

Medicine’s competence to set the norms defining illness, disease, disability and the ‘normal’ is the subject of many critiques. Daniel Goldberg’s paper on ‘railway spine’ further questions medicine’s ability, or willingness, to absorb narratives that are at odds with its own presumed objectivity. Railway spine refers to a late 19th century amalgam of symptoms suffered by individuals who had been involved in a railway accident. While pain was almost always a central feature, the diversity of patient experiences meant that doctors generally took a sceptical approach to the condition. Frequently, there were no ‘objective’ clinical findings and thus the subjective sufferings of the patient were discounted and put down to malingering. Goldberg’s argument is that we must use such historical lessons if we are to have any chance of alleviating the devastating burden of chronic pain stigma that persists today—stigma that results in epistemic and testimonial injustice.^18^

Like pain, voice hearing is an entirely subjective experience. Angela Woods interrogates the accounts of two young voice hearers through Dan Zahavi’s work on empathy and shame in order to open up new questions about the experience of hearing voices. As the paper points out, while there is no necessary correlation between hearing voices and suffering shame, there is a frequent coexistence. Woods suggests that using the notion of the ‘interpersonal self’, where the voice is ‘productive of the self’ (in contrast to the dominant conceptualisations of heard voices as either ‘independent of the self’ or ‘of the self’), might be fruitful as a mechanism for analysing the emotional experience of voice hearing.^19^

Shame frequently evokes a desire to hide, to conceal the source of shame and the emotion itself. Less commonly discussed than body shame, or shame experienced by patients, is the usually opaque matter of shame and the clinician. Deborah Bowman examines this theme through Nina Raine’s *Tiger Country*, a play that depicts the working lives of a group of doctors and nurses (of various levels of seniority) within a busy London hospital. Such institutions often have structures and hierarchies that systemically, and systematically, use shame as part of their organisational culture, and healthcare professionals survival responses aimed at protecting their professional identity may come at a significant cost: ‘Shame has a fundamental and overlooked relationship with damaging and well-documented phenomena in healthcare, including moral distress, ethical erosion, compassion fatigue, burnout, stress and ill health’.^20^

The importance of this may be better appreciated when viewed through the lens of Paul Gilbert’s paper contrasting shame and compassion. Gilbert’s extensive body of work on the subject identifies how shame experiences impact on our psychological selves, on how we experience ourselves, others and relationships. Shame serves to separate, segregate, marginalise and disengage. If the clinicians in Bowman’s account are so affected, then it would seem difficult for them to engage with what Gilbert sees as one antidote to shame—compassion. In contrast to shame, compassion—‘a sensitivity to suffering in self and others with a commitment to try to alleviate and prevent it’—facilitates connection, integration and support.^21^

Gilbert proposes that shame represents a major threat to human well-being as it increases vulnerability to illness, undermines physical and mental healing and reduces help seeking. Dolezal and Lyons take this as their starting point to explore shame’s impact on health. They argue that shame affects medical help seeking, disclosure, truthfulness, willingness to engage in treatment, cease health-affecting behaviours or return for further consultations. Furthermore, the pervasive use of shaming of ‘deviant’ bodies and behaviours in contemporary medical culture can inform how we view ourselves, and lead to the pernicious and damaging state of chronic shame. The stigmatising discourses surrounding minority groups (eg, age, race, sexuality) are degrading and powerful and also critical to health outcomes. Dolezal and Lyons call for broader political and clinical recognition of shame and stigma in respect of health and illness.^22^

The articles collected in this Special Issue consider a wide range of health-related topics—infectious disease, the dynamics of the clinical encounter, chronic pain, subjective experiences within mental health—thus demonstrating the far-reaching nature of shame and stigma. However, what is also evident from this Special Issue is just how little the role these factors play in medicine and healthcare has been subject to critical investigation. Much work, both empirical and conceptual, remains to be done in order to clarify the nature and extent of the burden of shame, the populations affected and the settings in which they are impacted on, and on the development and assessment of potential strategies aimed at mitigating problematic shame within healthcare contexts.

## References

[1] Verghese A Hope and clarity. The New York Times Magazine 22 2 2004 http://www.nytimes.com/2004/02/22/magazine/the-way-we-live-now-2-22-04-hope-and-clarity.html?mcubz=1 (accessed 17 Oct 2017).

[2] Nathanson DL Shame and pride: affect, sex, and the birth of the self. New York: WW Norton & Company, 1994.

[3] Sartre JP Being and nothingness: an essay on phenomenological ontology. London: Routledge, 2003.

[4] Sedgwick EK, Frank A, Shame and its sister: a silvan tompkins reader. Durham, NC: Duke University Press, 1995.

[5] Shaw GB Man and superman. London: Penguin, 2000.

[6] Lazare A Shame and humiliation in the medical encounter. Arch Intern Med 1987;147:1653–8. 10.1001/archinte.1987.00370090129021 3632171

[7] Ofri D What doctors feel: how emotions affect the practice of medicine. Boston: Beacon Press, 2013. 10.7899/JCE-14-23 PMC4211592 25222631

[8] Edson M Wit. New York: Dramatists play service, Inc, 1999.

[9] Shame and medicine project. http://www.shameandmedicineproject.com/

[10] Scheff TJ Elias, Freud and Goffman: Shame as the Master Emotion : Loyal S, Quilley S, The sociology of Norbert Elias. Cambridge: Cambridge University Press, 2004.

[11] Mendible M American shame: stigma and the body politic. Bloomington: Indiana University Press, 2016.

[12] Weiss MG, Ramakrishna J, Somma D Health-related stigma: rethinking concepts and interventions. Psychol Health Med 2006;11:277–87. 10.1080/13548500600595053 17130065

[13] Goffman E Stigma: notes on the management of spoiled identity. London: Penguin Books, 1990.

[14] Hutchinson P, Dhairyawan R Shame, stigma, HIV: philosophical reflections. Med Humanit 2017;43:225–30. 10.1136/medhum-2016-011179 28790137 PMC5739830

[15] Northrop JM A dirty little secret: stigma, shame and hepatitis C in the health setting. Med Humanit 2017;43:218–24. 10.1136/medhum-2016-011099 28363990

[16] Cock E ‘He would by no means risque his reputation’: patient and doctor shame in Daniel Turner’s *De Morbis Cutaneis* (1714) and *Syphilis* (1717). Med Humanit 2017;43:231–7. 28096307 10.1136/medhum-2016-011057 PMC5739831

[17] McLaughlin J The medical reshaping of disabled bodies as a response to stigma and a route to normality. Med Humanit 2017;43:244–50. 28167617 10.1136/medhum-2016-011065 PMC5739832

[18] Goldberg DS Pain, objectivity and history: understanding pain stigma. Med Humanit 2017;43:238–43. 10.1136/medhum-2016-011133 28228477

[19] Woods A On shame and voice-hearing. Med Humanit 2017;43:251–6. 10.1136/medhum-2016-011167 28389551 PMC5739828

[20] Bowman D Vulnerability, survival and shame in Nina Raine’s *Tiger Country* . Med Humanit 2017;43:264–8. 10.1136/medhum-2017-011354 29079608

[21] Gilbert P Shame and the vulnerable self in medical contexts: the compassionate solution. Med Humanit 2017;43:211–7. 10.1136/medhum-2016-011159 29030410

[22] Dolezal L, Lyons B Health-related shame: an affective determinant of health? Med Humanit 2017;43:257–63. 10.1136/medhum-2017-011186 28596218 PMC5739839

